# Fucoxanthin—An Antibacterial Carotenoid

**DOI:** 10.3390/antiox8080239

**Published:** 2019-07-24

**Authors:** Tomasz M. Karpiński, Artur Adamczak

**Affiliations:** 1 Department of Medical Microbiology, Poznań University of Medical Sciences, Wieniawskiego 3, 61-712 Poznań, Poland; 2 Department of Botany, Breeding and Agricultural Technology of Medicinal Plants, Institute of Natural Fibres and Medicinal Plants, Kolejowa 2, 62-064 Plewiska, Poland

**Keywords:** brown seaweeds, algal pigments, antibacterial activity, disk-diffusion method, micro-dilution method

## Abstract

Fucoxanthin is a carotenoid produced by brown algae and diatoms. This compound has several biological properties such as antioxidant, anti-obesity, anti-diabetic, anticancer, and antimicrobial activities. Unfortunately, until now the latter effect has been poorly confirmed. The aim of this study was an evaluation of fucoxanthin activity against 20 bacterial species. Antimicrobial effect of fucoxanthin was determined by using the agar disc-diffusion and micro-dilution methods. The studied carotenoid acted against 13 bacteria growing in aerobic conditions. It was observed to have a significantly stronger impact on Gram-positive than Gram-negative bacteria. Mean zones of growth inhibition (ZOIs) for Gram-positive bacteria ranged between 9.0 and 12.2 mm, while for Gram-negative were from 7.2 to 10.2 mm. According to the agar disc-diffusion method, the highest activity of fucoxanthin was exhibited against *Streptococcus agalactiae* (mean ZOI 12.2 mm), *Staphylococcus epidermidis* (mean ZOI 11.2 mm), and *Staphylococcus aureus* (mean ZOI 11.0 mm), and in the microdilution test towards *Streptococcus agalactiae* with the minimal inhibitory concentration (MIC) of 62.5 µg/mL. On the other hand, fucoxanthin was not active against strict anaerobic bacteria.

## 1. Introduction

**Figure 1:**
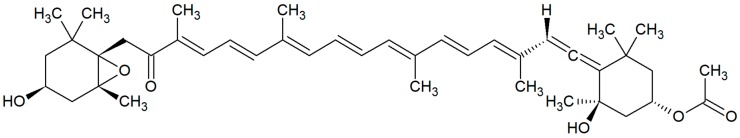
Chemical structure of fucoxanthin.

Edible seaweeds (red, brown and green marine macroalgae) are the rich source of various bioactive compounds: soluble dietary fibers, sulphated polysaccharides, phlorotannins, peptides, sulfolipids, polyunsaturated fatty acids, carotenoids, vitamins, and minerals [1]. Seaweed carotenoids mainly include: β-carotene, zeaxanthin, violaxanthin, lutein, and fucoxanthin [2]. Fucoxanthin is an orange-colored pigment predominantly found in brown algae (Phaeophyceae) and diatoms (Bacillariophyceae) [3,4]. It belongs to the class of xanthophylls and non-provitamin A carotenoids [5]. Its presence was confirmed, among others, in *Alaria crassifolia*, *Ascophyllum nodosum*, *Chaetoseros* sp., *Cladosiphon okamuranus*, *Cylindrotheca closterium*, *Cystoseira hakodatensis*, *Ecklonia stolonifera, Eisenia bicyclis*, *Fucus serratus*, *F. vesiculosus*, *Hijikia fusiformis*, *Himanthalia elongata*, *Ishige okamurae*, *Kjellmaniella crassifolia*, *Laminaria digitata*, *L. japonica, L. ochotensis*, *L. religiosa, Myagropsis myagroides*, *Odontella aurita*, *Padina tetrastromatica*, *Petalonia binghamiae*, *Phaeodactylum tricornutum*, *Sargassum fulvellum, S. heterophyllum*, *S. horneri*, *S. siliquastrum, Scytosiphon lomentaria, Sphaerotrichia divaricata, Turbinaria triquetra,* and *Undaria pinnatifida* [3,4,5,6,7,8,9,10,11,12,13,14]. Chemically, fucoxanthin is 3′-acetoxy-5,6-epoxy-3,5′-dihydroxy-6′,7′-didehydro-5,6,7,8,5′,6′-hexahydro-β,β-carotene-8-one with a molecular formula of C_42_H_58_O_6_ and a molecular weight of 658.906 g/mol [15]. This metabolite includes a unique allenic bond and some oxygenic functional moieties such as epoxide, hydroxyl, carbonyl, and carboxyl groups [3]. The chemical structure of fucoxanthin is shown in Figure 1.

It has recently been shown that fucoxanthin exhibits a lot of biological properties, including a protective activity against oxidative stress. It was presented that fucoxanthin prevents the cytotoxic effect of the oxidative agent in a dose-dependent manner and had a protective effect against UV-B radiation and DNA damaging factors [10,16,17,18,19]. This compound additionally affected the lipid metabolism and possesses anti-obesity and anti-diabetic activities [11,20,21]. The influence of fucoxanthin on the weight loss, insulin resistance, and the lowering of the blood glucose level were confirmed [22]. It also had a beneficial impact on the cardiovascular system, manifested by a decrease in cholesterol and triacylglycerol levels, the lowering of the blood pressure, and the reduction of inflammatory processes [23].

Additionally, fucoxanthin demonstrated broad anticancer activity. The antiproliferative effect was stated in vitro among others against the following cell lines: leukemic (HD-60) [24,25], epithelial colorectal adenocarcinoma (Caco-2, DLD-1 and HT-29) [26], prostate cancer (PC-3, DU-145, LNCaP) [27,28,29], urinary bladder cancer (EJ-1) [30], osteosarcoma (Saos-2, MNNG/HOS and 143B) [31], breast cancer (MDA-MB-231) [32], non-small-cell lung cancer (NSCLC) [33], and gastric adenocarcinoma (MGC-803) [34]. Other studies presented that fucoxanthin acted preventively against cancer exerting the antiangiogenic, antilymphangiogenic, and antimetastatic effects [32,35,36].

In the literature, there is mentioned about antibacterial properties of fucoxanthin [37,38]. Screening of the PubMed/MEDLINE database carried out at the beginning of June 2019 with the search term “fucoxanthin” found more than 600 items, however the combination of the keywords “fucoxanthin” and “antibacterial” gave only nine records. Unfortunately, none of these articles concerns the antibacterial activity of fucoxanthin. In the electronic databases, we found only three publications presenting studies in this field [7,8,12]. 

Due to the lack of research and not fully confirmed antibacterial properties of fucoxanthin, the aim of the present study was an evaluation of this carotenoid activity against selected clinical strains of bacteria.

## 2. Materials and Methods 

### 2.1. Microbial Strains and Culture Media

In this study, clinical strains of bacteria were used. None of the chosen strains was multi-resistant. Fucoxanthin was purchased from Sigma-Aldrich, Poland (product number: F6932, purity: ≥95%). Antimicrobial activity of this algal pigment was investigated against six Gram-positive bacteria (*Enterococcus faecalis*, *Staphylococcus aureus*, *S. epidermidis*, *Streptococcus agalactiae*, *S. pneumoniae*, and *S. pyogenes*), and seven Gram-negative bacteria (*Acinetobacter lwoffii*, *Escherichia coli*, *Klebsiella oxytoca*, *K. pneumoniae*, *Proteus mirabilis*, *Pseudomonas aeruginosa,* and *Serratia marcescens*) growing in aerobic conditions. The species of bacteria were grown at 35 °C for 24 h, in tryptone soy agar (TSA; Graso, Poland). Additionally, there were tested seven strict anaerobic bacteria (*Actinomyces israelii*, *Atopobium parvulum*, *Mitsuokella multacida*, *Peptococcus niger*, *Porphyromonas gingivalis*, *Propionibacterium acnes*, and *Veilonella parvula*). These species were cultured in anaerobic conditions using Genbox and Genbag anaer (bioMerieux, Poland) at 35 °C for 2–5 days, in Schaedler agar with 5% sheep blood (Graso, Poland). Two strains were tested for each species, except *A. israelii*, *M. multacida*, and *P. gingivalis* which were examined in one strain.

### 2.2. Antimicrobial Activity

The microbial growth inhibitory potential of the tested xanthophyll was determined by using the agar disc-diffusion method according to recommendations of the Clinical and Laboratory Standards Institute (CLSI) [39], and as described in our previous publication [40]. In brief, bacterial inocula of 0.5 McFarland were prepared. Next, 100 µL of all bacterial suspensions were inoculated on Mueller–Hinton agar with 5% sheep blood or Mueller–Hinton agar (Oxoid, Poland; Graso, Poland). Fucoxanthin was dissolved in 20% water solution of DMSO (Sigma-Aldrich, Poznań, Poland) in a final concentration of 1 mg/mL. A total of 25 µL of 1 mg/mL fucoxanthin (25 µg/disc) were transferred onto sterile filter papers (6 mm diameter). Additionally, sterile filter papers soaked 25 µL of 20% DMSO (negative control) were used. The plates were incubated at 35 °C for 18 h and anaerobes for two days. Results were shown as zones of growth inhibition (ZOIs). The experiments were repeated three times.

Minimal inhibitory concentration (MIC) was determined by the micro-dilution method using a 96-well plate (Nunc) according to CLSI [39]. Primarily, 100 µL of Mueller–Hinton broth, or Thioglicolate broth (Oxoid, Poland; Graso, Poland) for anaerobes, was placed in each well. The stock solution of fucoxanthin was transferred into the first well, and serial dilutions were performed so that concentrations in the range of 15.6 to 1000 µg/mL were obtained. The inoculums were adjusted to contain approximately 10^7^ CFU/mL bacteria. 10 µL of the proper inoculums were added to the wells. Additionally, 10 μL of 0.2% aqueous solution of 2,3,5-triphenyltetrazolium chloride (TTC) was added to each well. TTC is converted in bacterial cells into red, insoluble formazan crystals [41]. Next, the plates were incubated at 35 °C for 24 h. The MIC value was taken as the lowest concentration of the extract that inhibited any visible bacterial growth. The experiments were repeated three times.

### 2.3. Statistical Analysis 

The results reported in Table 1 are means ± SD of three parallel measurements, and medians. Data were tested using Statistica for Windows software. Statistical analysis of the results was based on Mann-Whitney U-test. Differences of *p* < 0.05 were considered to be significant.

## 3. Results

**Figure 2:**
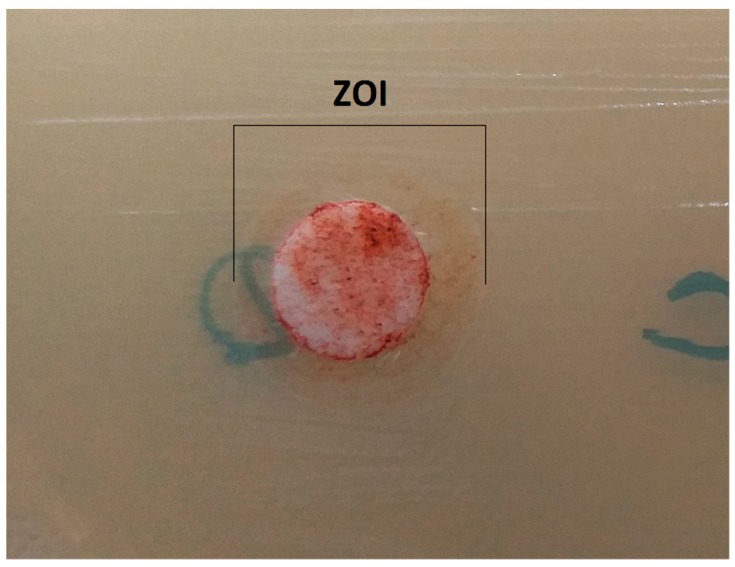
Culture of *Escherichia coli* strain. It is a visible zone of growth inhibition (ZOI) around the disk with 25 µg of fucoxanthin.

In the present research, the activity of fucoxanthin against bacterial strains belonging to 20 species was tested. This compound acted against 13 bacteria growing in aerobic conditions. It was observed a clearly stronger effect against Gram-positive than Gram-negative bacteria. Mean zones of growth inhibition (ZOIs) for Gram-positive bacteria were between 9.0 and 12.2 mm, while for Gram-negative ranged from 7.2 to 10.2 mm (Figure 2). The differences between the ZOIs of both groups were statistically significant (*p* < 0.0001). Minimal inhibitory concentrations (MICs) for Gram-positive bacteria reached values between 62.5 and 250 µg/mL (median 125 µg/mL), while for Gram-negative values were from 125 to 500 µg/mL (median 250 µg/mL). Between the MICs of both groups there were statistically significant differences (*p* = 0.0009). The highest activity of fucoxanthin in the agar disk-diffusion method was against *Streptococcus agalactiae* (mean ZOI 12.2 mm), *Staphylococcus epidermidis* (mean ZOI 11.2 mm), and *S. aureus* (mean ZOI 11.0 mm), and in the microdilution test towards *Streptococcus agalactiae* (MIC 62.5 µg/mL). Simultaneously, it was not found fucoxanthin’s activity against seven strict anaerobic bacteria. The obtained values of the ZOIs and MICs are presented in Table 1.

## 4. Discussion

In this paper, we presented the activity of fucoxanthin against 13 aerobic and 7 anaerobic bacteria. To our knowledge, this is the first work in which such a large number of species has been studied. Moreover, this is the first research of fucoxanthin, in which the minimal inhibitory concentrations (MICs) were tested. Fucoxanthin was investigated according to the disk-diffusion method at the concentration of 1 mg/mL. Additionally, it was used the microdilution assay in the levels of fucoxanthin from 15.6 to 1000 μg/mL. The results obtained in the second method amounted between 62.5 and 500 μg/mL. Deyab and Abou-Dobara [7] used this carotenoid isolated from the brown seaweed *Turbinaria triquetra* at the concentrations from 10 to 100 μg/mL. It can therefore be assumed that the used concentrations were similar. However, in our research the levels of the active compound were slightly higher. Unfortunately, in the above-mentioned article, the methodology of antimicrobial activity screening was not described, and the results were presented not according to the microbiological CLSI standards. These authors tested the activity of fucoxanthin against *E. coli*, *Bacillus cereus*, *B. subtilis*, *K. pneumoniae*, *S. aureus*, and *P. aeruginosa*. The zones of growth inhibitions for the above bacteria were 0.5–1.8 mm in the concentration of 10 μg/mL and 4.0–7.0 mm in 100 μg/mL. The ZOIs were very low and probably contrary to the guidelines for the disk-diffusion method, the width of the paper disk was not taken into account [7]. In our study, mean ZOIs amounted to 6.0 mm for anaerobic bacteria and from 7.2 to 12.2 mm for other species.

Rajauria and Abu-Ghannam [8] showed antimicrobial activity determined against *Listeria monocytogenes* using disc-diffusion method. The diameter of the growth inhibition zone reached 10.89 mm. The antimicrobial activity was demonstrated by both the purified fucoxanthin extracted from the brown alga *Himanthalia elongata* and chemical standard, at a concentration of 1 mg/mL (25 μg/disc). In our study, the same fucoxanthin concentration was used, and the ZOIs for tested bacteria were similar to this presented for *L. monocytogenes.*

Recently, the antibacterial properties of fucoxanthin were reported by Liu et al. [12]. This pigment was extracted from the edible seaweed *Undaria pinnatifida* with a purity of 82.70%, and tested against five human pathogens. According to the agar well diffusion method, fucoxanthin strongly inhibited the growth of Gram-positive bacteria: *B. subtilis*, *E. faecalis*, *S. aureus*, and *Enterococcus* sp. The diameters of their inhibition zones reached 25.49, 25.24, 21.80, and 12.66 mm, respectively. Similar to our results, they indicated weaker activity towards Gram-negative bacteria. In the case of *P. aeruginosa* it was 9.50 mm.

In the case of infection, mainly caused by Gram-negative bacteria, fucoxanthin may impact the reduction of inflammation. Gram-negative bacteria contain lipopolysaccharide (LPS), an endotoxin, which is a membrane component. During infection, LPS affects the inflammatory response, including septic shock, fever, and microbial invasion [42]. It was shown that fucoxanthin inhibited induced by LPS production of pro-inflammatory cytokines (IL-1β, IL-6, and TNF-α) by suppressing the NF-κB activation and the MAPK phosphorylation. Moreover, it reduced the levels of inducible nitric oxide synthase (iNOS) and cyclooxygenase 2 (COX-2) proteins [42,43,44]. Unfortunately, the direct antibacterial mechanism of fucoxanthin action is not known [12].

In the literature, there is a relationship between the antioxidant and antibacterial properties of natural chemical compounds [37]. The possible mechanisms of antibacterial activity of antioxidants include three basic ways: outer membrane permeability, cytoplasm leakage, and inhibition of nucleic acid formation [45]. The stronger effect of fucoxanthin against Gram-positive than Gram-negative bacteria, exhibited in our research and in the works of other authors [7,12], indicates that this biological activity depends on the differences in the cell wall structure and composition of both types of bacteria.

## 5. Conclusions

Our investigations confirm the antibacterial properties of fucoxanthin. The obtained results suggest that the above-mentioned substance can be a good antibacterial agent against some Gram-positive pathogens, including *Streptococcus agalactiae*, *Staphylococcus epidermidis*, *Staphylococcus aureus*, and weaker against Gram-negative ones (e.g., *Escherichia coli*, *Klebsiella oxytoca*, *K. pneumoniae*). On the other hand, it seems that fucoxanthin is not active towards strict anaerobic bacteria.

## Figures and Tables

**Table 1:** Antibacterial activity of fucoxanthin determined by the agar disc-diffusion and micro-dilution methods.

Studied Bacterial Strain	Zone of Growth Inhibition (ZOI) (mm)	Minimal Inhibitory Concentration (MIC) (µg/mL)
**Gram-positive**		
*Enterococcus faecalis*	9.0 ± 0.89	125–250
*Staphylococcus aureus*	11.0 ± 0.63	125
*Staphylococcus epidermidis*	11.2 ± 0.75	125
*Streptococcus agalactiae*	12.2 ± 0.75	62.5
*Streptococcus pneumoniae*	9.7 ± 0.52	125
*Streptococcus pyogenes*	10.0 ± 0.63	125
Mean of all ZOIs	10.5 ± 1.25	-
Median	10.0	125
**Gram-negative**		
*Acinetobacter lwoffii*	8.2 ± 0.41	250
*Escherichia coli*	10.2 ± 0.75	125
*Klebsiella oxytoca*	9.2 ± 0.75	125–250
*Klebsiella pneumoniae*	8.8 ± 0.75	250
*Proteus mirabilis*	7.2 ± 0.41	500
*Pseudomonas aeruginosa*	7.5 ± 0.55	250–500
*Serratia marcescens*	7.3 ± 0.52	500
Mean of all ZOIs	8.3 ± 1.18	-
Median	8.0	250
**Anaerobic**		
*Actinomyces israelii*	6.0	>1000
*Atopobium parvulum*	6.0	>1000
*Mitsuokella multacida*	6.0	>1000
*Peptococcus niger*	6.0	>1000
*Porphyromonas gingivalis*	6.0	>1000
*Propionibacterium acnes*	6.0	>1000
*Veilonella parvula*	6.0	>1000
**Negative control**		
20% DMSO	6.00 ± 0.00	-
